# Age, sex and *APOE*-ε4 modify the balance between soluble and fibrillar β-amyloid in non-demented individuals: topographical patterns across two independent cohorts

**DOI:** 10.1038/s41380-022-01436-7

**Published:** 2022-03-02

**Authors:** Raffaele Cacciaglia, Gemma Salvadó, José Luis Molinuevo, Mahnaz Shekari, Carles Falcon, Gregory Operto, Marc Suárez-Calvet, Marta Milà-Alomà, Arianna Sala, Elena Rodriguez-Vieitez, Gwendlyn Kollmorgen, Ivonne Suridjan, Kaj Blennow, Henrik Zetterberg, Juan Domingo Gispert, for the Alzheimer’s Disease Neuroimaging Initiative, for the ALFA study, Eider Arenaza-Urquijo, Annabella Beteta, Anna Brugulat-Serrat, Alba Cañas, Irene Cumplido, Carme Deulofeu, Ruth Dominguez, Maria Emilio, Karine Fauria, Sherezade Fuentes, José María González-de-Echavarri, Oriol Grau-Rivera, Laura Hernandez, Gema Huesa, Jordi Huguet, Iva Knezevic, Paula Marne, Carolina Minguillon, Tania Menchón, Maria Pascual, Albina Polo, Sandra Pradas, Gonzalo Sánchez-Benavides, Aleix Sala-Vila, Anna Soteras, Laia Tenas, Marc Vilanova, Natalia Vilor-Tejedor

**Affiliations:** 1 Barcelonaβeta Brain Research Center (BBRC), Pasqual Maragall Foundation, 08005 Barcelona, Spain; 2 Hospital del Mar Medical Research Institute (IMIM), 08005 Barcelona, Spain; 3 Centro de Investigación Biomédica en Red de Fragilidad y Envejecimiento Saludable (CIBERFES), 28089 Madrid, Spain; 4 Universitat Pompeu Fabra, 08002 Barcelona, Spain; 5 Centro de Investigación Biomédica en Red de Bioingeniería, Biomateriales y Nanomedicina (CIBERBBN), 28089 Madrid, Spain; 6 Servei de Neurologia, Hospital del Mar, Barcelona, Spain; 7 Department of Neurobiology, Care Sciences and Society, Division of Clinical Geriatrics, Center for Alzheimer Research, Karolinska Institutet, 141 52 Stockholm, Sweden; 8 Roche Diagnostics GmbH, Penzberg, Germany; 9 Roche Diagnostics International Lda, Rotkreuz, Switzerland; 10 Department of Psychiatry and Neurochemistry, Institute of Neuroscience and Physiology, The Sahlgrenska Academy at the University of Gothenburg, 41390 Mölndal, Sweden; 11 Clinical Neurochemistry Laboratory, Sahlgrenska University Hospital, 41390 Mölndal, Sweden; 12 UK Dementia Research Institute at UCL, WC1E 6BT London, UK; 13 Department of Neurodegenerative Disease, UCL Institute of Neurology, WC1N 3BG London, UK

**Keywords:** Neuroscience, Genetics, Biomarkers

## Abstract

Amyloid (Aβ) pathology is the earliest detectable pathophysiological event along the Alzheimer’s *continuum*, which can be measured both in the cerebrospinal fluid (CSF) and by Positron Emission Tomography (PET). Yet, these biomarkers identify two distinct Aβ pools, reflecting the clearance of soluble Aβ as opposed to the presence of Aβ fibrils in the brain. An open question is whether risk factors known to increase Alzheimer’s’ disease (AD) prevalence may promote an imbalance between soluble and deposited Aβ. Unveiling such interactions shall aid our understanding of the biological pathways underlying Aβ deposition and foster the design of effective prevention strategies. We assessed the impact of three major AD risk factors, such as age, *APOE*-ε4 and female sex, on the association between CSF and PET Aβ, in two independent samples of non-demented individuals (ALFA: *n* = 320, ADNI: *n* = 682). We tested our hypotheses both in candidate regions of interest and in the whole brain using voxel-wise non-parametric permutations. All of the assessed risk factors induced a higher Aβ deposition for any given level of CSF Aβ42/40, although in distinct cerebral topologies. While age and sex mapped onto neocortical areas, the effect of *APOE*-ε4 was prominent in the medial temporal lobe, which represents a target of early tau deposition. Further, we found that the effects of age and *APOE*-ε4 was stronger in women than in men. Our data indicate that specific AD risk factors affect the spatial patterns of cerebral Aβ aggregation, with *APOE*-ε4 possibly facilitating a co-localization between Aβ and tau along the disease *continuum*.

## Introduction

Amyloid (Aβ) pathology is thought to be among the earliest events occurring along the Alzheimer’s *continuum*, which is later followed by tau spread and cerebral atrophy [1]. Both cerebrospinal fluid (CSF) Aβ concentrations and Positron Emission Tomography (PET) with specific tracers, provide established biomarkers of Aβ pathology, and have shown considerable agreement, both on prognostic and diagnostic levels [2–4]. Yet, CSF and PET assays measure two different Aβ pools reflecting two distinct biological processes. While the former indexes the current status of soluble Aβ production *versus* clearance, the latter quantifies the presence of Aβ fibrillar plaques in the brain [5, 6]. It has been suggested that cumulative cerebral Aβ deposition observed in AD might stem from a dysregulation between the production and clearance of Aβ, and that Aβ plaques may act as a “sink”, hindering the transport of soluble Aβ fragments from the brain to the CSF [7]. In this respect, the study of factors affecting the balance between soluble and deposited Aβ may help identifying the underlying mechanisms promoting cerebral Aβ aggregation for any given level of CSF Aβ dysmetabolism. With this in mind, we investigated the impact of risk factors for Alzheimer’s dementia, such as *APOE*-ε4 genotype [8], older age [9] and female sex [10] on the relationship between CSF and PET markers of Aβ. We hypothesized that distinct risk factors may exacerbate cerebral Aβ accumulation, assessed by Aβ PET, after accounting for the level of soluble Aβ dysmetabolism, assessed by CSF Aβ42/40 concentrations, in specific cerebral topological patterns. In addition, to aid our understanding of putatively different underlying mechanisms, we tested the interactions between each of the risk factors and CSF Aβ40, the most abundant Aβ isoform found in the CSF, which has been regarded as a marker of global protein clearance, rather than an index of pathology [11]. With this respect, some studies found moderate but significant increases of CSF Aβ40 in Aβ-PET positive compared with negative participants [12] as well as in AD patients compared with asymptomatic individuals [13, 14]. This suggests that abnormalities in both production and clearance of Aβ peptide may result in a net accumulation of fibrillar Aβ in the brain. We reasoned that if a significant interaction with CSF Aβ40 is found, that would be supportive of a deficient mechanism in total Aβ clearance, given that Aβ40 does not tend to aggregate in fibrillar plaques [15].

We tested our hypotheses in regions of vulnerability to AD proteinopathy and further examined the whole-brain using a voxel-wise approach, on a monocentric cohort of middle-aged cognitively unimpaired (CU) participants (ALFA sample). Furthermore, we repeated all analyses in an independent sample of non-demented participants derived from the Alzheimer’s Disease Neuroimaging Initiative (ADNI), which included CU individuals along with participants with mild cognitive impairment (MCI), in order to extend the analysis further in the Alzheimer’s *continuum*.

## Methods

### Study participants

The ALFA study (Clinicaltrials.gov Identifier: NCT01835717) comprises a longitudinal monocentric research platform aiming at the identification of pathophysiological alterations in preclinical AD. The ALFA cohort is composed of 2,743 CU individuals, all reporting a Clinical Dementia Rate score of 0, most of them being first-order descendants of AD patients [16]. Within this research framework, the ALFA+ is a nested study that includes advanced imaging protocols, including magnetic resonance imaging (MRI) and PET acquisitions, along with cognitive, lifestyle factors as well as fluid biomarkers. The present study included the first 320 consecutive participants of the ALFA+ study with available CSF, Aβ PET, MRI and cognitive data. None of the subjects had a neurologic or a psychiatric diagnosis. All the tests and image acquisitions were measured within less than a year time-difference. The ALFA+ study (ALFA-FPM-0311) was approved by the Independent Ethics Committee “Parc de Salut Mar,” Barcelona, and registered at Clinicaltrials.gov (Identifier: NCT02485730). All participating subjects and signed the study’s informed consent form that had also been approved by the Independent Ethics Committee “Parc de Salut Mar,” Barcelona. The study was conducted according to the Declaration of Helsinki. The confirmation cohort included all CU and MCI ADNI participants, with available Aβ CSF, Aβ PET and MRI data acquired within less than one year, resulting in a sample of 682 individuals. ADNI is a multi-site open access dataset designed to accelerate the discovery of biomarkers to identify and track AD pathology (http://adni.loni.usc.edu/). Data collection and sharing in ADNI (Clinicaltrials.gov Identifier: NCT00106899) were approved by the Institutional Review Board of each participating institution, and written informed consent was obtained from all participants. For both cohorts, we provide the mini mental state examination test score, as a measure of dementia screening tool (MMSE) [17].

Procedures for *APOE* genotype are described in the Supplementary Materials.

### CSF sampling and analysis

For ALFA participants, CSF samples were obtained by lumbar puncture following standard procedures [18] (please refer to the Supplementary Material for details on CSF sampling). Aβ40 as well as Aβ42 concentrations were determined with the NeuroToolKit (Roche Diagnostics International Ltd.) on cobas Elecsys e601 (Aβ42) and e411 (Aβ40) instruments at the Clinical Neurochemistry Laboratory, University of Gothenburg, Sweden. CSF collection and analyses for ADNI participants are described in the ADNI procedure manual (http://adni.loni.usc.edu/methods/). Aβ40 and Aβ42 concentrations in ADNI were measured with 2D-UPLC-tandem mass-spectrometry at the University of Pennsylvania. To increase sensitivity, both in ALFA and ADNI the ratio between Aβ42 and Aβ40 was finally calculated [12].

### PET imaging acquisition procedures

Imaging procedures from ALFA have been described previously [19]. In brief, Aβ PET images were acquired 90 min post-injection using [^18^F]flutemetamol with 4 frames of 5 min each. A T1-weighted 3D-TFE sequence was acquired with a 3 T Philips Ingenia CX scanner with the following sequence parameters: voxel size = 0.75 mm isotropic, field of view (FOV) = 240 × 240 × 180 mm^3^, flip angle = 8°, repetition time = 9.9 ms, echo time = 4.6 ms, TI = 900 ms. Details of ADNI imaging procedures can also been found in the website (http://adni.loni.usc.edu/methods/documents/). In brief, [^18^F]florbetapir Aβ PET images were acquired in four frames of five minutes each, 50–70 min post-injection. Finally, structural MRI data were acquired on 3T scanning platforms using T1-weighted sagittal 3-dimensional magnetization-prepared rapid-acquisition gradient echo sequences (MP-RAGE). Image preprocessing is described in the Supplementary Materials.

### Regional Aβ-PET quantification

SUVRs were extracted from a-priori defined regions of interest (ROI). We selected the cortical Centiloid composite ROI (http://www.gaain.org/centiloid-project) as Aβ-sensitive cerebral region [20]. As tau-vulnerable regions, we selected the Braak stages ROIs [21] defined according to the Desikan–Killiany atlas (DK atlas) in Schöll et al. [22]. Supplementary Fig. 1 shows both the Centiloid and Braak stages ROIs mapped onto the DK atlas. For visualization purposes, the Centiloid ROIs was parceled onto the DK atlas according to a best-match visual criterion. Supplementary Table 1 shows the full list of the DK atlas labels that were used for both composite ROIs.

### Statistical analyses

Demographic information from both cohorts was compared using *t*-test for continuous variables and Chi-squared test for categorical ones.

For our main objective, we first looked for interactions between CSF Aβ42/40 concentrations and each of the three assessed AD risk factors (i.e., age, sex and *APOE*-ε4 status), in promoting cerebral Aβ deposition in regions that are selectively vulnerable to either Aβ (Centiloid composite ROI) or tau pathology (Braak stages ROIs). This first set of analyses was conducted with the SPSS software package (https://www.ibm.com/analytics/spss-statistics-software). Next, we conducted a spatially unbiased whole-brain analysis to detect interaction effects in distributed brain areas. This was achieved by performing a voxel-wise non-parametric inference using randomize in the FMRIB Software Library v6.0 (https://fsl.fmrib.ox.ac.uk/fsl/fslwiki/), using threshold-free cluster enhancement (TFCE) [23] with 5000 permutations. For both the ROI and whole-brain analyses, we set-up three different general linear models where Aβ PET was set as dependent variable, while CSF Aβ42/40, age, sex and *APOE*-ε4 status were modeled as predictors. The interaction term involving CSF Aβ42/40 and any of the three AD risk factors was modeled as independent variable, as follows:

$$A\beta \,PET = CSF\,A\beta + age + sex + APOE\varepsilon 4 + CSF\,A\beta \ast APOE\varepsilon 4$$

$$A\beta \,PET = CSF\,A\beta + age + sex + APOE\varepsilon 4 + CSF\,A\beta \ast age$$

$$A\beta \,PET = CSF\,A\beta + age + sex + APOE\varepsilon 4 + CSF\,A\beta \ast sex$$

To avoid muticollinearity, continuous CSF Aβ42/40 values were centered to the group mean [24]. *APOE*-ε4 was treated as categorical binary variable (i.e., 0 = non-carriers, 1 = ε4-carriers). In all statistical models, age was treated as continuous variable. In addition, we investigated whether the interactions between CSF Aβ42/40 and *APOE*-ε4 as well as age returned significantly different result in men and women. To this aim, we repeated the above-mentioned analyses stratifying by sex. For the ROI analyses, results were considered significant if surviving a threshold of *p* < 0.05 corrected for multiple testing using a False-Discovery Rate (FDR) approach. In FSL, we set t-contrasts on the interaction terms, testing for putative significant interactions between CSF Aβ42/40 concentrations and each of the assessed risk factors, in both directions. For these analyses, results were considered significant if surviving a threshold of *p* < 0.05 corrected for multiple testing with a family-wise error rate correction (FWE). All the above-mentioned statistical models were applied to the ADNI replication sample, with the inclusion of clinical diagnosis, defined as a binary categorical variable (0 = cognitively unimpaired, 1 = MCI), as a covariate. Statistical models for assessing the main effects of each risk factor, as well as CSF Aβ42/40, are described in the Supplementary Materials.

## Results

### Sample characteristics

Demographic characteristics of both cohorts can be found in Table 1. Of the 682 ADNI participants, 423 (62%) had a clinical diagnosis of MCI, including early, typical and late MCI. Compared to ALFA, ADNI participants were significantly older, more educated, and harbored a lower proportion of *APOE*-ε4 carriers. In addition, the ALFA cohort hosted a significantly higher proportion of women, compared with ADNI. Table 1 additionally includes the proportion of Aβ positive individuals based on both CSF and PET data, for both cohorts. Cut-off values for both of these measures are reported in the Supplementary Materials.

**Table 1:** Sample characteristics.

	ALFA (*n* = 320)	ADNI (*n* = 682)	*p* value
Age, M(SD)*	61.11 (4.57)	72.64 (7.12)	<0.001
Age range, year	49.99–73.64	55.10–93.80	
Education, M(SD)*	13.41 (3.52)	16.21 (2.62)	<0.001
Female sex, *n* (%)	200 (62.5%)	329 (48.2%)	0.001
*APOE*-ε4, *n* (%)	170 (53.12%)	279 (39.58%)	<0.001
*CSF Aβ42/40 positive*, *n* (%)	110 (34.4%)	370 (54.25%)	<0.001
Centiloid range	−23.88–81.63	−28.92–169.70	<0.001
A*β* PET positive, *n* (%)**	50 (15.6%)	423 (62.02%)	<0.001
MMSE, *M(SD)*	29.17 (0.96)	28.43 (1.62)	0.002

*MMSE* Mini-mental state examination test.

^a^Expressed in years.

^b^Based on Centiloid data.

### ROI analyses

Supplementary Table 2 shows the results of each statistical model run for the different risk factors in each of the tested ROIs; for each model, the F-statistic and the FDR correct *p* value of the interaction term are presented. Within the Centiloid ROI, we observed a significant interaction between CSF Aβ42/40 and age in both cohorts, while the interaction with sex was only significant in ALFA participants. By contrast, the interaction involving *APOE*-ε4 was significant in ADNI but not in ALFA. In Braak I/II ROIs, we found a significant interaction between CSF Aβ42/40 and *APOE*-ε4 in both cohorts even though in ADNI the nominally significant value (p=0.037) did not reach survive FDR correction, while the interactions with age and sex were not significant. In Braak III/IV ROIs, all interaction models were significant in both cohorts except for that involving CSF Aβ42/40 and sex, which was not significant in ADNI. Finally, in Braak V/VI ROIs, we observed significant interactions between CSF Aβ42/40 and age in both cohorts, while the one with sex was significant only in in ALFA, and no significant interactions were found in either cohort involving *APOE*-ε4.

All of the above-mentioned effects indicate that, for a given level of CSF Aβ42/40 concentrations, *APOE*-ε4, age, and female sex promoted a higher cortical Aβ deposition in the designated cerebral areas. Supplementary Table 3 shows the results of interaction models with CSF Aβ40. In ALFA, only the interaction with age were significant, and specifically in the Centiloid, Braak III/IV, and Braak V/VI ROIs. By contrast, in ADNI no significant interactions were found, in any ROIs.

### Whole-brain analysis

#### Main effects

Supplementary Fig. 2 shows the main effects of our predictors on Aβ PET, in both cohorts. As expected, CSF Aβ42/40 concentrations were negatively related to Aβ PET uptake in widespread cortical areas. Age and *APOE*-ε4 were positively associated to cortical Aβ deposition, while the effects of sex were marginal (data not shown). Finally, we found a circumscribed yet significant effect of CSF Aβ40 on PET uptake in ALFA, and ADNI, in the thalamus and superior temporal areas.

#### Interaction effects

**Fig. 1:**
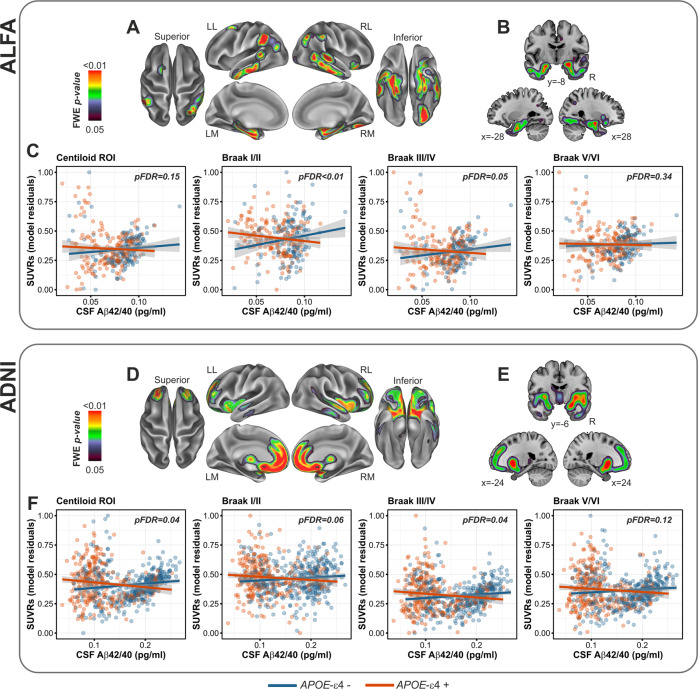
*APOE*-ε4 significantly modified the spatial topography of Aβ PET as function of CSF Aβ42/40. **A**, **B** Surface and volume rendering in ALFA participants of the Aβ PET statistical probability map resulting from the interaction model. Compared to non-carriers, *APOE*-ε4 carriers displayed higher SUVRs, for any given level of CSF Aβ42/40, in medial temporal regions including entorhinal cortex and hippocampus. **C** Group scatterplots in ALFA participants showing the significant interaction between *APOE*-ε4 and CSF Aβ42/40 in a priori defined progressive ROIs. **D**, **E** Surface and volume rendering in ADNI participants, of Aβ PET statistical probability map indicating that compared to non-carriers, *APOE*-ε4 carriers displayed higher SUVRs, for any given level of CSF Aβ42/40, in right inferior and middle temporal as well as right insula. **F** Group scatterplots in ADNI participants showing the significant interaction between *APOE*-ε4 and CSF Aβ42/40 in a priori defined progressive ROIs. LL Left lateral, LM Left medial, RL Right lateral, RM Right medial.

The interaction between CSF Aβ42/40 and *APOE*-ε4 was significant in both cohorts in a highly symmetrical pattern involving medial temporal lobe areas including the anterior hippocampus and the inferior temporal cortex, as well as the orbitofrontal cortex. In ALFA, this interaction further mapped onto the bilateral entorhinal cortex (Fig. 1a–c), while in ADNI there was an involvement of additional areas such as the insular cortex, the orbitofrontal gyrus and the caudate nuclei (Fig. 1d–f).

**Fig. 2:**
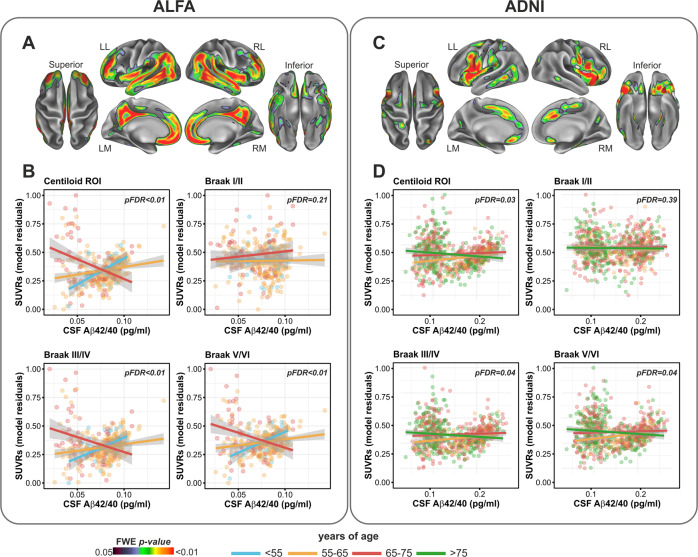
Age significantly modified the association between of CSF Aβ42/40 and Aβ PET. **A**, **B** In ALFA participants, older individuals displayed, for any given level of CSF Aβ42/40 concentration, a higher Aβ PET retention in distributed cerebral areas including inferior and superior temporal cortex as well as medial prefrontal and inferior parietal areas. Group scatterplot show these interactions in a priori defined progressive ROIs. **C**, **D** Interaction was replicated in the ADNI cohort, although in a less distributed topological pattern. For visualization purposes, age continuous variable was broken down in four subgroups. LL Left lateral, LM Left medial, RL Right lateral, RM Right medial.

Similarly, the interaction between CSF Aβ42/40 and age was significant in both cohorts, although in a different spatial patters with respect to the interaction with *APOE*-ε4. Specifically, this interaction mapped onto distributed neocortical areas including the anterior, middle and posterior cingulate cortex as well as inferior parietal, middle temporal and insular cortices in both cohorts, even though in ADNI the effects sizes were generally lower and the topology for this interaction was less widespread (Fig. 2a–d).

**Fig. 3:**
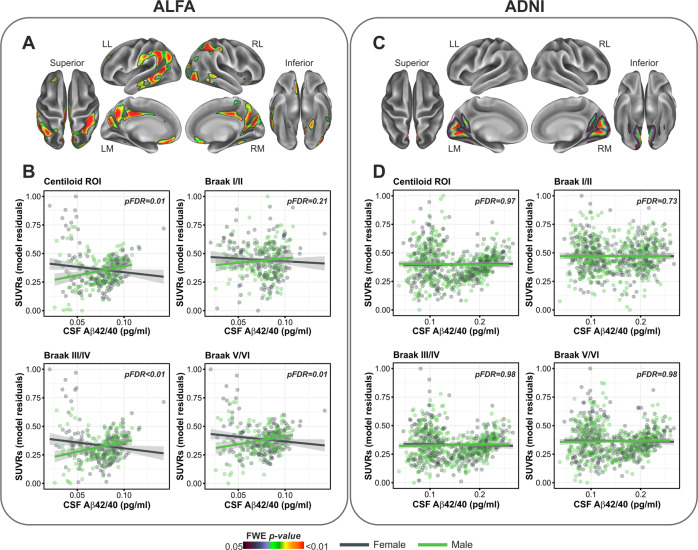
Sex significantly modified the association between CSF Aβ42/40 and Aβ PET. **A**, **B** In ALFA participants, sex significantly modulated the association between CSF Aβ42/40 and Aβ PET uptake indicating higher SUVRs in females women compared to men, in posterior medial regions including the precuneus and the cuneus. Group scatterplot show these interactions in a priori defined progressive ROIs. **C**, **D** An overlapping cortical topology was found in ADNI participants indicating the same interaction effects as in ALFA. LL Left lateral, LM Left medial, RL Right lateral, RM Right medial.

Finally, we observed a significant, yet less spatially extended, interaction in both groups between CSF Aβ42/40 and sex, indicating a higher PET signal in women compared to men, for a given level of CSF Aβ42/40 concentrations, in posterior middle cortical regions (Fig. 3a–d).

To rule out that the topological patterns resulting from these interactions may be due to exceptionally low amount of fribrillar Aβ, and therefore driven by noise, we performed a post-hoc sensitivity analysis in the ALFA cohort, by repeating those interactions in the subsample of Aβ-positive individuals, defined based on their CSF Aβ42/40 concentrations. The results of these analyses are consistent with that observed in the entire cohort (please refer to the Supplementary Materials).

When assessing the interactions between each of the three risk factors and CSF Aβ40, in line with our previously shown ROI data, we found a significant interaction with age in the ALFA cohort mapping onto a distributed set of brain regions, indicating that, in older individuals, higher CSF Aβ40 concentrations reflected into higher cortical SUVRs (Supplementary Fig. 3). No significant interactions between CSF Aβ40 and *APOE*-ε4 or sex were found. Finally, in ADNI, there was no significant interaction between CSF Aβ40 and any of the assessed risk factors.

### Analyses stratified by sex

**Fig. 4:**
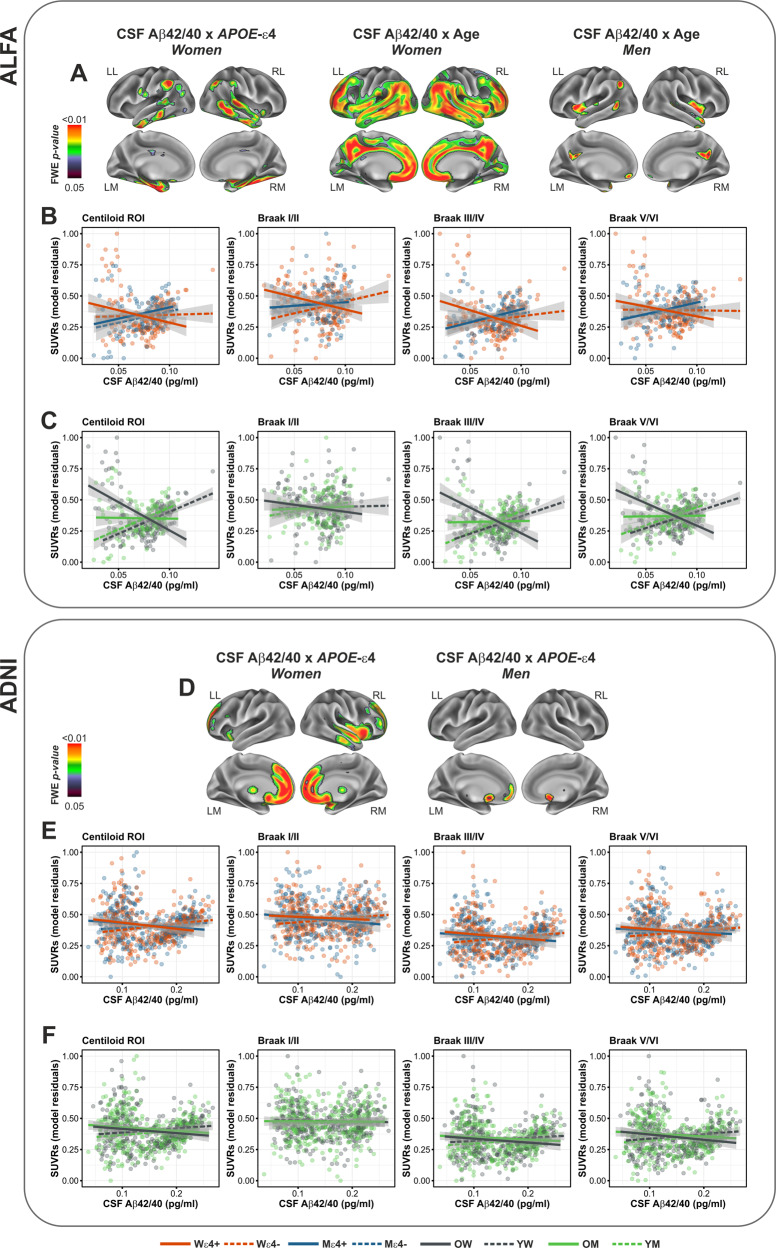
Analyses stratified by sex. **A** Surface rendering of *p* value maps, for the interaction between CSF Aβ42/40 and *APOE*-ε4, as well as age, separately for women and men in the ALFA sample. **B**, **C** Group scatterplots, stratified by sex, of the assessed interactions in regions of interest. **D** Same as in **A**, in the ADNI sample. **E**, **F** Same as in **B** and **C**, in the ADNI sample. *Wε4+* Women *APOE*-ε4 carriers, *Wε4−* Women *APOE*-ε4 non-carriers, *Mε4+* Men *APOE*-ε4 carriers, *Mε4−* Men *APOE*-ε4 non-carriers.

In both samples, we found a significantly more widespread results in women than in men. Specifically, the interaction between CSF Aβ42/40 and *APOE*-ε4 did not return significant results in men in the ALFA sample (Fig. 4A, B), and only a small cluster in the caudate in ADNI (Fig. 4D, E). Similarly, the interaction with age, was more prominent in females than in males, in both samples (Fig. 4A, C, D, F). This indicates that the previous findings reported in the whole sample were largely driven by women compared to men. Supplementary Table 3 shows the results for each of the interaction models, computed separately for men and women, in our selected ROIs.

Confirmatory 3-way interactions performed in both samples indicate that the modulatory effects of age and *APOE*-ε4 were significantly larger in females compared with males, although the result involving *APOE*-ε4 reached statistical significance only in the ALFA cohort (Supplementary Figs. 5 and 6).

## Discussion

The present work aimed to determine whether unmodifiable risk factors for AD modulate the association between soluble and deposited Aβ species quantified with CSF concentrations and PET imaging, respectively. We tested our hypotheses on two independent cohorts of non-demented participants across distinct phases of the Alzheimer’s *continuum*. We found that *APOE*-ε4, older age and female sex, resulted in a higher fibrillary plaques deposition for any given level of CSF Aβ42/40, with each risk factor mapping onto a specific topology. Similar, although not identical, patterns were observed in both cohorts for the same risk factors. Notably, the two cohorts differed in the PET tracers used for Aβ imaging and CSF Aβ sampling, as well as in the average age and level of progression in the Alzheimer’s *continuum*, thus reinforcing the robustness and generalizability of our results.

In both cohorts, we observed significant interactions between CSF Aβ42/40 and *APOE*-ε4 in Braak stages I/II and III/IV ROIs, although in ADNI the former interaction only reached a nominal significance level (*p* = 0.037). Whole-brain analyses conducted in the ALFA cohort confirmed that, compared to non-carriers, *APOE*-ε4 carriers displayed, for any given value of CSF Aβ42/40, higher Aβ PET retention in a symmetric pattern covering medial temporal lobe (MTL) areas and including the anterior hippocampus, parahippocampus, entorhinal cortex, inferior temporal as well as the bilateral inferior parietal regions. Similarly, in ADNI this interaction covered the bilateral anterior hippocampus and the inferior temporal cortex, with the inclusion of additional areas such as the insular cortex, the orbitofrontal gyrus and the caudate nuclei.

These data suggest that *APOE*-ε4 carriership may modify the patterned spreading of cerebral Aβ accumulation as a function of early Aβ dysmetabolism and point to the MTL as a vulnerable region for the incipient Aβ accumulation, in those individuals harboring the genetic risk. Importantly, MTL regions do not typically display Aβ accumulation in the early stages of the disease, which rather involve neocortical areas and particularly prefrontal cortex, posterior cingulate, precuneus, and inferior parietal, as shown by in-vivo staging [25–27] and autopsy studies [21, 28]. On the other hand, the MTL displays selective vulnerability to early tau deposition, as previously documented in patients along the Alzheimer’s *continuum* [29–31], *as* well as in CU individuals [22, 32]. According to a disease model of Aβ-induced tau hyperphosphorylation, fibrillary Aβ initiates a pathophysiological cascade leading to tau misfolding that eventually propagates throughout the neocortex [33, 34]. One study reported that the interaction between Aβ and tau in driving a greater risk of developing AD, mapped onto inferior temporal and parietal regions [35], which overlap with the regions we found in both cohorts. Hence, our results suggest that *APOE*-ε4, by exacerbating cortical Aβ deposition in MTL areas, might facilitate the spread of tau in extra medial-temporal regions, plausibly promoting an earlier co-localization of Aβ and tau. In line with this, previous PET imaging studies have documented a higher tau deposition in *APOE*-ε4 carrier AD patients compared to non-carriers [29, 36–38]. Furthermore, our interaction effects may help to explain the faster disease progression [39–41] as well as the stronger relationship between Aβ and cognitive decline [42, 43] in *APOE*-ε4 carriers compared to non-carriers.

We next reported a significant modulatory effect for age in driving a higher Aβ PET uptake as a function of CSF Aβ42/40. This interaction was significant in brain areas typically subject to Aβ deposition (i.e., the Centiloid ROI), in both cohorts. Interestingly, in the ALFA cohort, this effect was also observed for the interaction between age and CSF Aβ40, thus suggesting that an age-related decline in the clearance of overall soluble Aβ species from the brain might underlie the higher levels of Aβ deposition observed with more advanced age, for any given level of CSF Aβ42/40. In ADNI, the significant interaction between CSF Aβ42/40 and age was also found, even though in a less extended topology and lower effect sizes. Such a reduced effect in ADNI compared to that in ALFA may be due to the different age range of the two samples (ALFA = 50-73; ADNI = 56–94 years). In fact, earlier studies indicate that, the effects of aging on cortical Aβ deposition drop significantly in individuals older than 60 years of age [44], which also may explain the non-significant result when we assessed the interaction between CSF Aβ40 and age in ADNI. That interaction was however significant in the ALFA cohort, suggesting a failure in the efficiency of Aβ clearance as putative candidate underlying mechanism, rather than an over production of Aβ42. The lack of a significant interaction between CSF Aβ40 and *APOE*-ε4 status suggests, by contrast, that *APOE*-ε4 carriers may be subject to an over-production of Aβ42 oligomers in MTL areas, in *APOE*-ε4 carriers compared to non-carriers. This interpretation is further supported by the evidence that CU *APOE*-ε4 carriers show hippocampal over-activation during memory tasks [45, 46], which may in turn favor a higher Aβ42 production over time, given that Aβ42 is released concomitantly with neural activity [47]. Hence, one possibility is that MTL hyper-activation may occur at the cost of higher Aβ42 production over time in *APOE*-ε4 carriers. Even though it is well acknowledged that *APOE*-ε4 relates to a deficient Aβ clearance [8], our findings are now suggesting that these individuals might additionally be subject to a higher production of Aβ42 specifically in the medial MTL and inferior temporal regions.

Finally, we reported that the interaction with sex was significant in the Centiloid ROI, as well as Braak stages ROIs III/IV and V/VI in the ALFA cohort, while no significant interactions involving sex were retrieved in the ADNI sample using an ROI approach. Whole-brain analyses yielded a significant interaction in posterior medial regions such as the posterior cingulate cortex and cuneus, as well as middle temporal areas in ALFA, while a less distributed effect was found in ADNI, involving the cuneus bilaterally. The lack of such a replication in the ADNI sample, however, may be substantiated by several factors. First, the ALFA cohort harbored a significantly higher proportion of women, compared to ADNI. Second, the effect of sex in promoting a higher risk for AD pathology may be further mitigated by age, and particularly being prominent during the perimenopause, a transitional phase occurring in midlife (i.e., 40 and 60 years of age), characterized by estrogen depletion with consequent loss of neuroprotective functions [48]. Perimenopause is characterized by increased variability in terms of neurological symptoms, which is restored later in life, presumably due to a new endocrine homeostasis [49]. Consistent with this, we observed a significant modulatory role of sex in the balance between Aβ dysmetabolism and Aβ aggregation, in a cohort of middle-aged individuals (ALFA) but not in a sample more advanced in age (ADNI). Our stratified analyses additionally indicate that the deleterious effects of *APOE*-ε4 and age in prompting a higher regional SUVRs as a function of CSF Aβ42/40 concentrations, is remarkably higher in women compared to men, suggesting that the three risk factors may further interact among each other to exacerbate the emergence and progression of AD pathology. Specifically, women may be more aggravated by harboring the *APOE*-ε4 allele than males, particularly when still completely asymptomatic and in their middle age (ALFA cohort), as also suggested previously [50]. This is reflected by the significant interaction we observed in women but not in men, between CSF Aβ42/20 and *APOE*-ε4, only in the ALFA sample. By contrast, the combined effects of age and sex over the unbalance of soluble vs deposited Aβ, may extend beyond this asymptomatic stage and persist in later stages of pathology (ADNI sample).

One limitation of our study consists in the use of a linear model to explain the association between CSF and PET Aβ markers, which is known for being not linear [2–6]. To mitigate this risk, we used non-parametric statistics in our unbiased voxel-wise approach, which confirmed the findings of the ROI-based analyses. The adoption of non-linear statistical modeling may improve the proportion of the variance explained by this association. Second, the cross-sectional design concomitantly with the lack of tau PET prevents us from confirming that *APOE*-ε4 carriers may display higher tau spread in the MTL for a given level of Aβ dysmetabolism. In addition, future studies shall include a longitudinal assessment of neuropsychological data, to determine how an imbalance between soluble and aggregated Aβ impacts cognitive performance in multiple domains.

In summary, our strategy of assessing the impact of risk factors on the association between two distinct surrogate markers of cleared and aggregated Aβ provide novel insights into the biological pathways underlying Aβ aggregation in the brain. Moreover, these data clarify the mechanisms underlying the higher AD prevalence associated to those risk factors.

## Supplementary information

Supplementary Materials

## Data Availability

Data used in preparation of this article were obtained from the Alzheimer’s Disease Neuroimaging Initiative (ADNI) database (adni.loni.usc.edu). As such, the investigators within the ADNI contributed to the design and implementation of ADNI and/or provided data but did not participate in analysis or writing of this report.
