# Supplementary material for: Age, sex and APOE-ε4 modify the balance between soluble and fibrillar β-amyloid in non-demented individuals: topographical patterns across two independent cohorts
Source: Mol Psychiatry. 2022 Mar 2;27(4):2010–8. doi: 10.1038/s41380-022-01436-7 (PMC9126807; doi:10.1038/s41380-022-01436-7)
Supplement: Supplementary file 1 — Supplementary Materials [file 41380_2022_1436_MOESM1_ESM.docx]

**Title:** Age, sex and *APOE*-ε4 modify the balance between soluble and fibrillar β-amyloid in non-demented individuals: topographical patterns across two independent cohorts

**Authors:**

Raffaele Cacciaglia^*^, Gemma Salvadó^*^, José Luis Molinuevo, Mahnaz Shekari, Carles Falcon, Gregory Operto, Marc Suárez-Calvet, Marta Milà-Alomà, Arianna Sala, Elena Rodriguez-Vieitez, Gwendlyn Kollmorgen, Ivonne Suridjan, Kaj Blennow, Henrik Zetterberg, and Juan Domingo Gispert, for the Alzheimer’s Disease Neuroimaging Initiative, for the ALFA study

*These two authors contributed equally

**Supplementary Materials**

**CSF sampling procedures in the ALFA cohort**

CSF was collected into a 15mL sterile polypropylene sterile tube (Sarstedt. Nümbrecht. Germany; cat. no. 62.554.502). CSF was aliquoted in volumes of 0.5mL into sterile polypropylene tubes (0.5mL Screw Cap Micro Tube Conical Bottom; Sarstedt. Nümbrecht. Germany; cat. no. 72.730.005). and immediately frozen at −80°C. Overall. the time between collection and freezing was less than 30 minutes. All the determinations were done in aliquots that had never been previously thawed.

**Cut-off values for CSF and PET-based amyloid**

In ALFA, participants were classified as Aβ positive if their CSF Aβ42/40 value was below 0.071 (**Milà-Alomà et al., 2020**), while for ADNI cut-off value was set to 191 (assessed through CSF Aβ42 mass spectrometry) (**Shaw et al., 2009**). Centiloid cut-off for both cohorts was set to 12 (**Salvadò et al., 2019**).

***APOE genotype***

For ALFA participants, total DNA was obtained from blood cellular fraction by proteinase K digestion followed by alcohol precipitation. For ADNI, DNA was extracted by Cogenics from a 3-mL aliquot of EDTA blood ([adni.loni.usc.edu/data-samples/genetic-data/](file:///C:\Users\Raffaele\Documents\materiali_CSF_PET_paper\adni.loni.usc.edu\data-samples\genetic-data\)). Both samples were genotyped for two single nucleotide polymorphisms (SNPs), rs429358 and rs7412 to define the *APOE*-ε2, ε3 and ε4 alleles. For both cohorts, subjects were classified as ε4 carriers (one or two alleles) or non-carriers. Out of the 320 study participants in ALFA, 21 were ε2/ε3, 129 were ε3/ε3, 8 were ε2/ε4, 135 were ε3/ε4, and 27 were ε4/ε4. In ADNI, 58 were ε2/ε3, 345 were ε3/ε3, 10 were ε2/ε4, 219 were ε3/ε4, and 50 were ε4/ε4. In all analyses involving *APOE*-ε4 status as factor, participants were divided in two groups, being non-carriers (*i.e.*, ε2/ε3 + ε3/ε3) and carriers (ε2/ε4 + ε3/ε4 + ε4/ε4).

***Image preprocessing***

For both cohorts, individual PET frames were co-registered to produce a mean image, which was subsequently spatially registered onto the respective structural MRI scan. Afterwards, the new segment function in SPM12 was employed to segment gray matter from MRI scans, which were normalized to the Montreal Neurological Institute (MNI) space. along with the PET images. We calculated the standardized uptake value ratio (SUVR) in MNI space using the whole cerebellum as reference region. Prior to statistical analysis images were smoothed with an 8-mm full width at half-maximum (FWHM) Gaussian kernel.

***Supplementary statistical analyses***

In both the ALFA and ADNI cohorts, main effects of cerebrospinal fluid (CSF) Aβ42/40, age, sex, and *APOE*-ε4 status on Aβ-PET uptake were assessed by setting up a voxel-wise linear regression in SPM12 (<https://www.fil.ion.ucl.ac.uk/spm/> ), with the normalized and smoothed standardized uptake value ratio (SUVR) individual parametric maps entered as dependent variables, while modeling the above mentioned factors as independent explanatory variables. Results were considered significant if surviving a voxel-level threshold of p<0.001 applying a cluster extent correction of 100 contiguous voxels (k > 100).

To assess the combined effects of AD risk factors, we performed additional analyses testing three-way interactions involving CSF Aβ42/40 and each pair of the tested risk factors. To this aim, we set up three different statistical models where the effects of CSF Aβ42/40 on Aβ PET were studied in combination with either *APOE*-ε4 and age, *APOE*-ε4 and sex, or age and sex.

$$A\beta PET=CSF A\beta+age+sex+APOE\varepsilon4+CSF A\beta*APOE\epsilon4*age$$

$$A\beta PET=CSF A\beta+age+sex+APOE\varepsilon4+CSF A\beta*APOE\epsilon4*sex$$

$$A\beta PET=CSF A\beta+age+ sex+APOE\varepsilon4+CSF A\beta*age*sex$$

***Supplementary results***

*Main effects*

*Main effect of CSF Aβ42/40. age and APOE-ε4 on cortical amyloid deposition*

In both cohorts. Continuous CSF Aβ42/40 was strongly and negatively associated to Aβ-PET uptake in widespread regions across the cortical mantle known to be target of early Aβ deposition (Chételat et al.. 2013). Age was significantly related to a higher cortical Aβ deposition, even though the effect was much more prominent and widespread in ALFA. compared to ADNI. The effects of sex were marginal, and mostly indicating a higher deposition in women compared to men in midline cortical areas including anterior and middle cingulate cortex. in both cohorts. There was no significant effect of *APOE*-ε4 on to Aβ-PET retention, however, removing CSF Aβ42/40 from the model resulted in a significant main effect of the ε4 allele, indicating a higher Aβ deposition in carrier vs. non-carriers. across the cingulate cortex as well as in superior temporal areas (Fig. S2).

*Sensitivity analysis in the ALFA cohort*

We performed a sensitivity analysis assessing the interactions between CSF Aβ42/40 and each of the three risk factors (*i.e,* age, *APOE*-ε4, sex) in the subgroup of Aβ-positive, in the ALFA cohort (defined as CSF Aβ42/40 < 0.071, as in Milà-Alomà et al., 2020). This subsample consisted of n=110 individuals with a mean age of 62.16 years (SD=5.06). For this analysis, we applied a more liberal threshold of p<0.005 on the voxel level, due to the reduced number of subjects, masking the results with a binary region of interest taken from the data collected in the whole sample. The results of these analyses are consistent with observed in the entire cohort, thus suggesting that the interactions yielded in the whole sample do not tap into noise (Fig. S4).


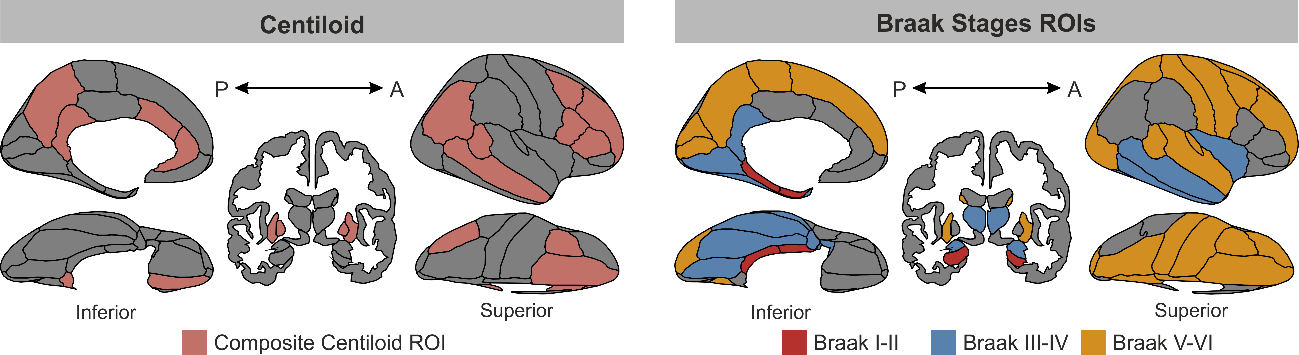


**Fig. S1 Regions of interest projected onto the Desikan-Killiany atlas.** Representation of both cortical and subcortical structures included in the Centiloid and Braak stages ROIs. Polygon rendering is created with the “ggseg” package in R (<https://github.com/LCBC-UiO/ggseg/tree/master>).

**
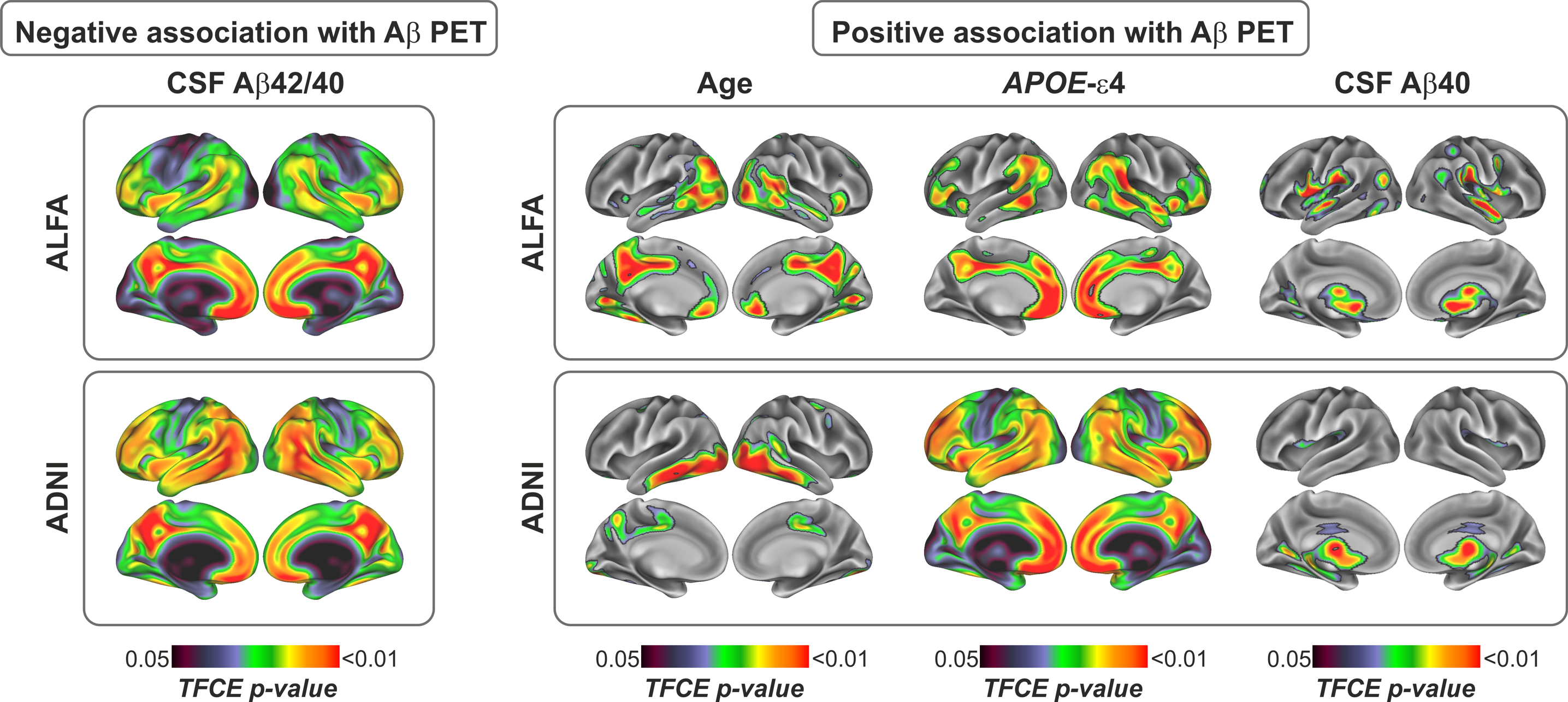
**

**Fig. S2.** Main effects of CSF Aβ42/40, age, *APOE*-ε4, and CSF Aβ40 on Aβ PET tracer retention in both cohorts


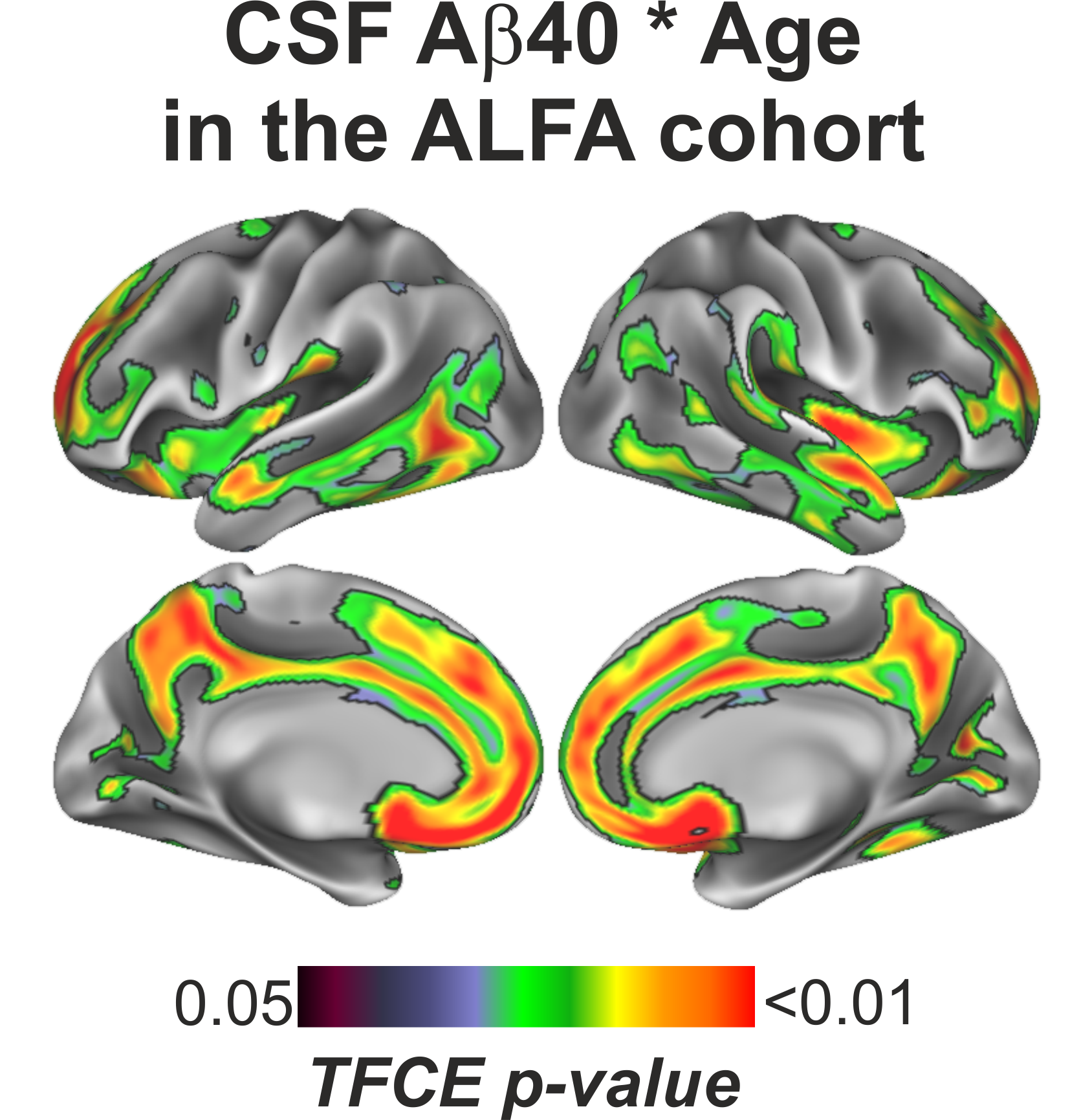


**Fig. S3.** Interaction between CSF Aβ40 and age in the ALFA cohort over Aβ PET data, indicating that, with older age, the linear association between CSF Aβ40 and Ab PET uptake becomes positive.


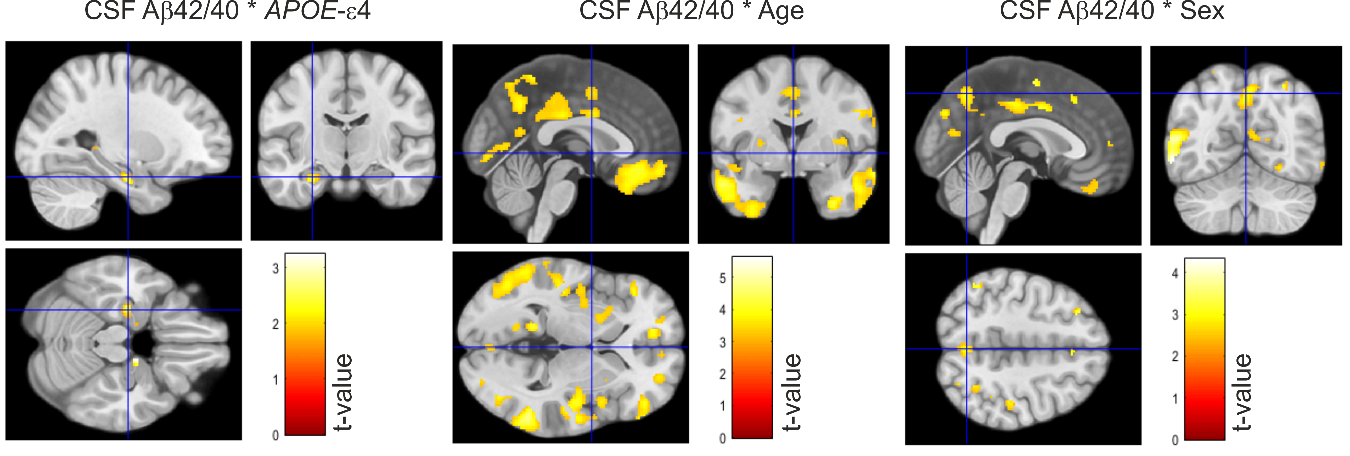


**Fig. S4.** Sensitivity analysis conducted in Ab-negative individuals in the ALFA cohort shows consistent results as in the entire ALFA sample

**
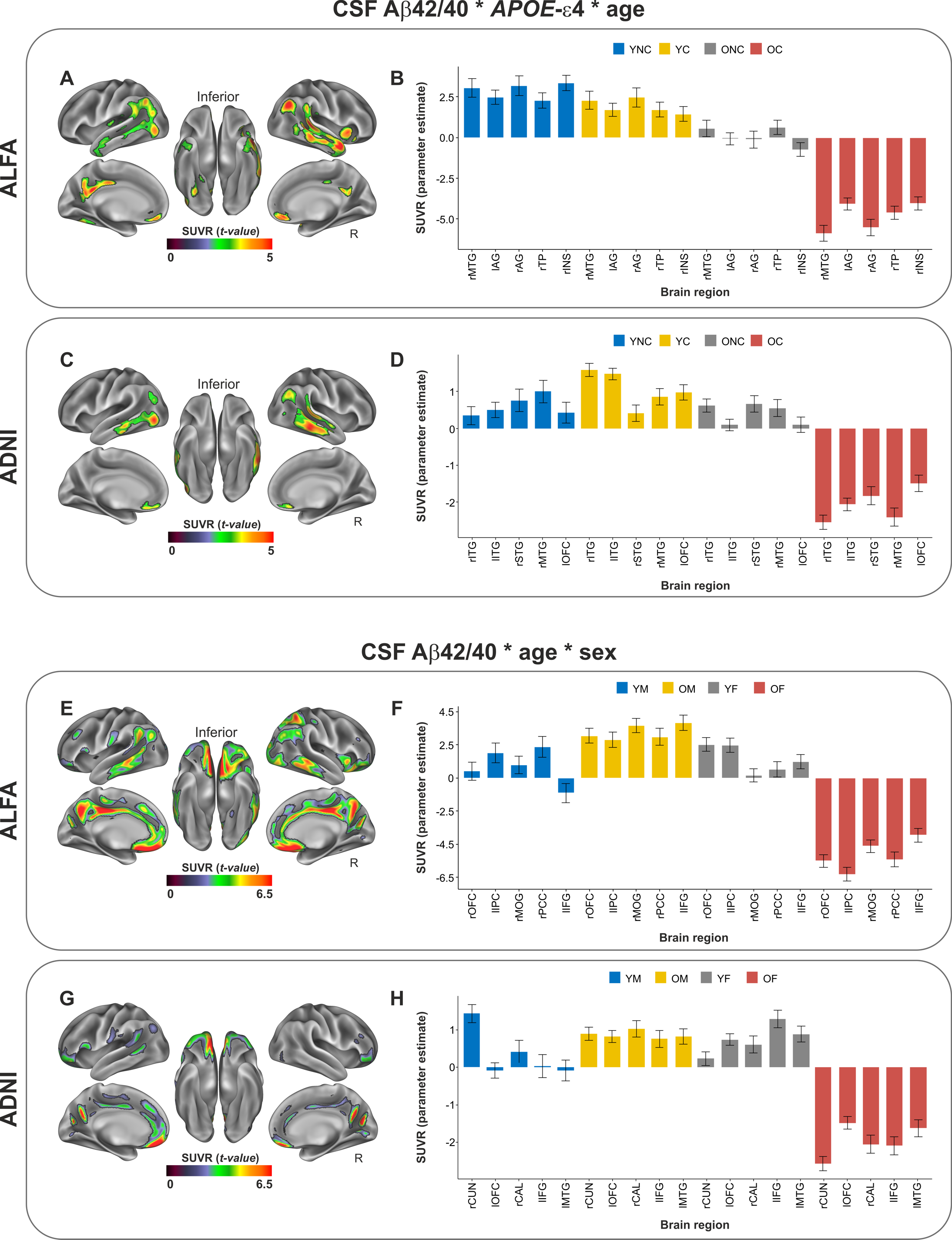
**

**Fig. S5. Three-way interactions in both cohorts. A-B)** Surface rendering and bar plots indicating three-way interactions involving CSF Aβ42/40, *APOE*-ε4 and age on Aβ PET uptake in the ALFA cohort. YNC=young non-carriers; YC=young ε4-carriers; ONC=older non-carriers; rMTG=right middle temporal gyrus; lAG=left angular gyrus; rAG=right angular gyrus; rTP=right temporal pole. **C-D)** Same as in A-B, in the ADNI cohort. rINS=right insula; rITG=right inferior temporal gyrus; lIPG=left inferior parietal gyrus; rSTG=right superior temporal gyrus; lOFC=orbitofrontal cortex. In B) and D), bars in the plot encode the interaction between one categorical (*APOE*-ε4) and two continuous (CSF Aβ42/40, age) variables. **E-F)** Surface rendering and bar plots indicating three-way interactions involving CSF Aβ42/40, age and sex on Aβ PET uptake in the ALFA cohort. YM=young males; OM=older males; YF=young females; OF=older females; rOFC=right orbitofrontal cortex; lIPC=left inferior parietal cortex; rMOG=right medial orbital gyrus; rPCC=right posterior cingulate cortex; lIFG=left inferior frontal gyrus; **G-H)** Same as in E-F, in the ADNI cohort. rCUN=right cuneus; lOFC=left orbitofrontal cortex; rCAL=right calcarine; lIFG=left inferior frontal gyrus; lMTG=left middle temporal gyrus. In F) and H), bars in the plot encode the interaction between one categorical (*s*ex) and two continuous (CSF Aβ42/40, age) variables

**
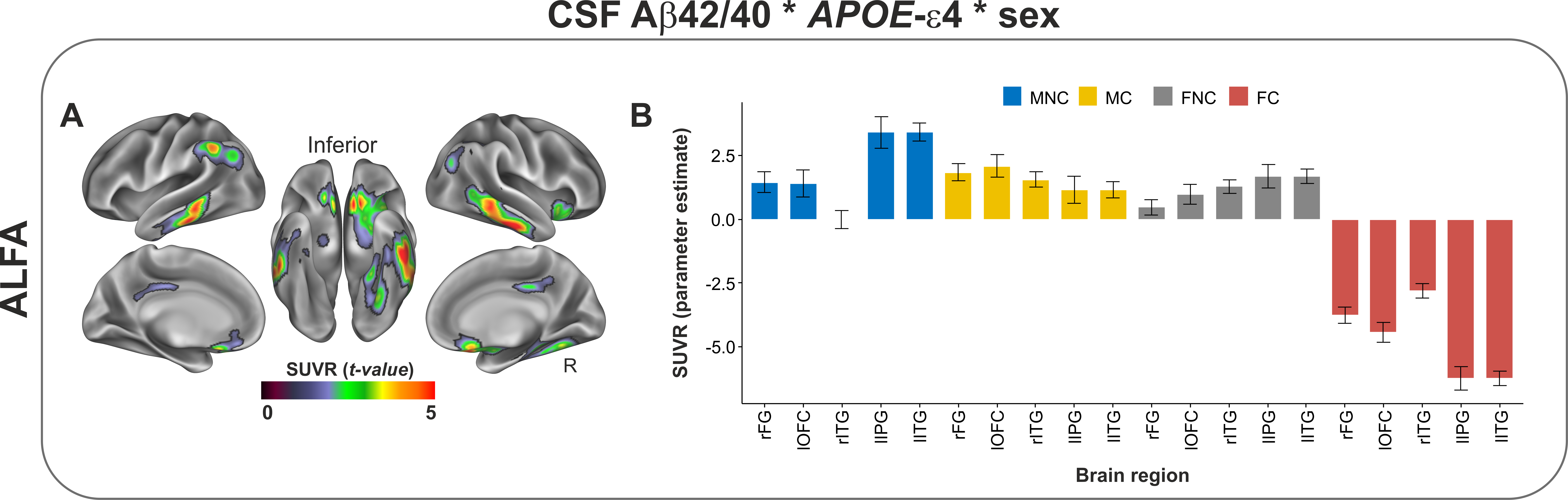
**

**Fig. S6 Three-way interactions in the ALFA cohort.**  **A-B)** Surface rendering and bar plots indicating three-way interactions involving CSF Aβ42/40, age and sex on Aβ PET uptake in the ALFA cohort. MNC=males non-carrier; MC=males ε4-carriers; FNC=females non-carrier; FC=females ε4-carriers; FG=right fusiform gyrus; lOFC=orbitofrontal cortex; rITG=right inferior temporal gyrus; lIPG=left inferior parietal gyrus; lITG=left inferior temporal gyrus. Bars in the plot encode the interaction between two categorical (*APOE*-ε4, sex) and one continuous (CSF Aβ42/40) variables.

**Supplementary Table 1** – List of Desikan-Killiany labels included in Braak stages and composite Centiloid ROIs

|  |  | **Desikan-Killiany labels** |
| --- | --- | --- |
| **Centiloid** |  |  |
|  |  | Rostral middle frontal; Caudal middle frontal; Pars triangularis; Pars opercularis; Inferior parietal; Banks of superior temporal sulcus; middle temporal; Rostral anterior cingulate; Caudal anterior cingulate; Isthmus cingulate; Medial orbitofrontal; Putamen. Pallidum. |
| **Braak stage** |  |  |
|  | I-II | Entorhinal cortex; Hippocampus |
|  | III-IV | Fusiform; Lingual; Amygdala; Inferior temporal; Middle temporal; Temporal pole; Thalamus; Isthmus cingulate; Insula |
|  | V-VI | Superior frontal; Paracentral; Precuneus; Cuneus; Pericalcarine; Rostral middle frontal; Caudal middle frontal; Precentral; Postcentral. Superior parietal; Lateral occipital; Transverse temporal; Banks of superior temporal sulcus; Superior temporal; Caudate; Putamen |

**Supplementary Table 2 – Interactions between CSF Aβ42/40 and each AD risk factor over Aβ-PET uptake in vulnerable ROIs**

|  | **Centiloid ROI** | | | | **Braak I/II** | | | | **Braak III/IV** | | | | **Braak V/VI** | | | |
| --- | --- | --- | --- | --- | --- | --- | --- | --- | --- | --- | --- | --- | --- | --- | --- | --- |
|  | **ALFA** | | **ADNI** | | **ALFA** | | **ADNI** | | **ALFA** | | **ADNI** | | **ALFA** | | **ADNI** | |
|  | F_1,314_ | pFDR | F_1,675_ | pFDR | F_1,314_ | pFDR | F_1,675_ | pFDR | F_1,314_ | pFDR | F_1,675_ | pFDR | F_1,314_ | pFDR | F_1,675_ | pFDR |
| CSFAβ_42/40_**APOE-ε4* | 2.43 | 0.15 | 8.57 | **0.04** | 9.29 | **<0.01** | 4.38 | **0.06** | 4.49 | 0.05 | 6.37 | **0.04** | 0.89 | 0.34 | 3.18 | 0.12 |
| CSFAβ_42/40_*age | 45.18 | **<0.01** | 6.01 | **0.03** | 1.71 | 0.21 | 1.26 | 0.39 | 34.97 | **<0.01** | 6.07 | **0.04** | 34.55 | **<0.01** | 6.52 | **0.04** |
| CSFAβ_42/40_*sex | 7.01 | **0.01** | 0.58 | 0.97 | 1.83 | 0.21 | 0.35 | 0.73 | 8.70 | **<0.01** | 0.04 | 0.98 | 6.81 | **0.01** | 0.01 | 0.98 |

**Supplementary Table 3 – Interactions between CSF Aβ40 and each AD risk factor over Aβ-PET uptake in vulnerable ROIs**

|  | **Centiloid ROI** | | | | **Braak I/II** | | | | **Braak III/IV** | | | | **Braak V/VI** | | | |
| --- | --- | --- | --- | --- | --- | --- | --- | --- | --- | --- | --- | --- | --- | --- | --- | --- |
|  | **ALFA** | | **ADNI** | | **ALFA** | | **ADNI** | | **ALFA** | | **ADNI** | | **ALFA** | | **ADNI** | |
|  | F_1,314_ | pFDR | F_1,675_ | pFDR | F_1,314_ | pFDR | F_1,675_ | pFDR | F_1,314_ | pFDR | F_1,675_ | pFDR | F_1,314_ | pFDR | F_1,675_ | pFDR |
| CSFAβ_40_**APOE-ε4* | 0.99 | 0.48 | 0.66 | 0.81 | 0.01 | 0.98 | 0.06 | 0.90 | 0.62 | 0.57 | 0.49 | 0.88 | 0.42 | 0.61 | 0.91 | 0.63 |
| CSFAβ_40_*age | 16.44 | **<0.01** | 0.01 | 0.99 | 4.47 | 0.09 | 0.44 | 0.85 | 15.16 | **<0.01** | 0.06 | 0.99 | 15.26 | **<0.01** | 0.07 | 0.98 |
| CSFAβ_40_*sex | 1.52 | 0.36 | 0.19 | 0.85 | 0.12 | 0.78 | 0.03 | 0.95 | 1.58 | 0.48 | 0.15 | 0.89 | 1.60 | 0.41 | 0.22 | 0.99 |

**Supplementary Table 4 – Analyses stratified by sex in a-priori defined regions of interest**

|  | **Centiloid ROI** | | | | **Braak I/II** | | | | **Braak III/IV** | | | | **Braak V/VI** | | | |
| --- | --- | --- | --- | --- | --- | --- | --- | --- | --- | --- | --- | --- | --- | --- | --- | --- |
|  | **ALFA** | | **ADNI** | | **ALFA** | | **ADNI** | | **ALFA** | | **ADNI** | | **ALFA** | | **ADNI** | |
|  | F | pFDR | F | pFDR | F | pFDR | F | pFDR | F | pFDR | F | pFDR | F | pFDR | F | pFDR |
| **In men** |  |  |  |  |  |  |  |  |  |  |  |  |  |  |  |  |
| CSFAβ**APOE-ε4* | 0.01 | 0.97 | 1.93 | 0.31 | 0.20 | 0.77 | 1.87 | 0.31 | 0.41 | 0.77 | 1.81 | 0.31 | **0.19** | 0.77 | 0.42 | 0.58 |
| CSFAβ*age | 4.99 | 0.11 | 1.91 | 0.31 | 0.59 | 0.77 | 0.02 | 0.89 | 5.67 | 0.11 | 1.64 | 0.31 | 2.55 | 0.29 | 1.41 | 0.31 |
|  |  |  |  |  |  |  |  |  |  |  |  |  |  |  |  |  |
| **In women** |  |  |  |  |  |  |  |  |  |  |  |  |  |  |  |  |
| CSFAβ**APOE-ε4* | 5.13 | 0.03 | 8.15 | 0.04 | 13.40 | <0.01 | 2.52 | 0.13 | 11.26 | <0.01 | 5.04 | 0.06 | 3.49 | 0.07 | 4.13 | 0.07 |
| CSFAβ*age | 52.93 | <0.01 | 3.80 | 0.07 | 1.32 | 0.25 | 2.11 | 0.14 | 35.49 | <0.01 | 4.64 | 0.06 | 45.30 | <0.01 | 5.69 | 0.064 |

**Supplementary References**

Chetelat G. La Joie R. Villain N. Perrotin A. de La Sayette V. Eustache F*. et al.* Amyloid imaging in cognitively normal individuals. at-risk populations and preclinical Alzheimer's disease. NeuroImage Clinical 2013; 2: 356-65.

Mila-Aloma M, Salvado G, Gispert JD, Vilor-Tejedor N, Grau-Rivera O, Sala-Vila A *et al.* Amyloid beta, tau, synaptic, neurodegeneration, and glial biomarkers in the preclinical stage of the Alzheimer's continuum. *Alzheimer's & dementia : the journal of the Alzheimer's Association* 2020; **16**(10)**:** 1358-1371.

Rodrigue KM. Kennedy KM. Devous MDS. Rieck JR. Hebrank AC. Diaz-Arrastia R. et al. β-Amyloid burden in healthy aging: regional distribution and cognitive consequences. Neurology 2012; 78: 387–395.

Salvadó G, Molinuevo JL, Brugulat-Serrat A, Falcon C, Grau-Rivera O, Suarez-Calvet M *et al.* Centiloid cut-off values for optimal agreement between PET and CSF core AD biomarkers. *Alzheimer's research & therapy* 2019; 11(1): 27.

Shaw LM, Vanderstichele H, Knapik-Czajka M, Clark CM, Aisen PS, Petersen RC *et al.* Cerebrospinal fluid biomarker signature in Alzheimer's disease neuroimaging initiative subjects. *Annals of neurology* 2009; **65**(4)**:** 403-413.
